# Climate, health and disease

**DOI:** 10.4103/0972-2327.41872

**Published:** 2008

**Authors:** Sanjeev V. Thomas

**Affiliations:** Editor, Annals of Indian Academy of Neurology, Department of Neurology, Sree Chitra Tirunal Institute for Medical Sciences and Technology, Trivandrum - 695 011, India. E-mail: sanjeev@sctimst.ac.in

This issue of the Annals of Indian Academy of Neurology (AIAN) will be reaching the readers when several North Indian states would be reeling under heat waves. At the same time, Monsoon rains would be ravaging the states in South India. The theme for this year's World Health Day-April 7, 2008-is ‘Protecting health from climate change’. Two months later, on June 5, 2008, the World Environment Day would be celebrated with the slogan ‘Kick the Habit! Towards a low carbon economy’. The Norwegian Nobel Peace Committee awarded the Nobel Peace Prize for 2007 to the Intergovernmental Panel on Climate Change and Mr. Al Gore Jr. in recognition of their efforts to build up and disseminate greater knowledge concerning the climate change brought about by the activities of humans, and to lay the foundations for the measures required for counteracting such change. Global warming is threatening to bring about extraordinary changes in our beautiful planet-The Earth. How do these changes influence human health, and particularly the nervous system? Climate, environment and health are closely interrelated. A change in one would necessarily disturb the rest and modify the health and disease state of populations at macro and micro levels. Climatic change can modify the incidence of several neurological disorders. Multiple sclerosis (MS) was considered to be a disease of the temperate climate, but MS is observed quite frequently in tropical countries too. Several vector-borne diseases tend to aggravate as a result of change in climate and environment. The World Health Organization (WHO) has identified five areas in which climate changes would impair human health. It can lead to failure of agriculture which in turn may aggravate food scarcity. It is anticipated that deaths occurring due to malnutrition would escalate from the current staggering figure of 3.5 million deaths per year. Experts in the field have shown that changes in climate can alter the microbial systems, disease transmission patterns, water and agro ecosystem, socioeconomic and demographic milieu of the society. These changes in turn have a profound impact on health due to an increase in disease and death due to temperature and extreme weather-related diseases, air pollution, water- and foodborne diseases, vector- and rodent-borne diseases, food and water shortage, mental, infectious and nutrition-related diseases. WHO has recognized the need for research in order to develop methods for breaking this chain of events. We, as practicing neurophysicians, need to be aware of these changes and their implications on human health.

In this issue, we have a review article regarding inherited metabolic disorders. These disorders had never received their rightful position in the curriculum of modern medicine. It is estimated that 1 in 1000 live born infants have inherited metabolic disorders. In our country, most of these disorders present in forme fruste and are never diagnosed, so much so that doctors are under the erroneous impression that inherited metabolic disorders are rare. Another misconception is that these disorders present only in childhood. Recently, there has been considerable progress in the diagnosis and treatment of several of these disorders. Bone Marrow or cord blood transplant has become a successful treatment option for mucopolysacharidoses and possibly several leukodystrophies. Acid alpha-glucosidase enzyme replacement has now become an approved treatment for infantile Pompe disease that presents with fatal myopathy and cardiomyopathy. Several deaths have been averted and ventilator-free life had become possible for these children who otherwise would have died in 24 months or so. The advantage of intravenous replacement of this enzyme in adolescent patients is currently under trial. Regrettably, in India, there is no facility for the screening and diagnosis of most of the inherited metabolic disorders. With the advent of a new technology of tandem mass spectrometry, it is now possible to simultaneously screen for more than 25 inherited metabolic disorders by using a 3 mm blood spotted on a filter paper. Several developed countries have set up routine screening program for identifying newly born infants affected at the time of birth in order to minimize the adverse clinical effects and disability. It is the right of every infant born in this country to have access to appropriate laboratory services to ascertain his or her status with regard to inherited metabolic disorders. We need to set up a national program for routine screening for metabolic disorders at birth. Indian Academy of Neurology and other scientific forums engaged in health care need to impress the Government to set up such facilities in all the states in this country.

